# Trends in utilisation and inequality in the use of reproductive health services in Sub-Saharan Africa

**DOI:** 10.1186/s12889-019-7865-z

**Published:** 2019-11-21

**Authors:** Gordon Abekah-Nkrumah

**Affiliations:** Department of Public Administration and Health Services Management, University of Ghana Business School, P. O. Box 78, Legon, Accra, Ghana

**Keywords:** State, Health, Inequalities, Sub-Saharan Africa

## Abstract

**Background:**

The paper argues that unlike the income literature, the public health literature has not paid much attention to the distribution of substantial improvements in health outcomes over the last decade or more, especially, in the Sub-Saharan African (SSA) context. Thus, the paper examines current levels of utilisation, changes in utilisation as well as inequality in utilisation of reproductive health services over the last 10 years in SSA.

**Methods:**

The paper uses two rounds of Demographic and Health Survey (DHS) data from 30 SSA countries (latest round) and 21 countries (earlier round) to compute simple frequencies, cross-tabulated frequencies and concentration indices for health facility deliveries, skilled delivery assistance, 4+ antenatal visits and use of modern contraceptives.

**Results:**

The results confirm the fact that utilisation of the selected reproductive health services have improved substantially over the last 10 year in several SSA countries. However, current levels of inequality in the use of reproductive health services are high in many countries. Interestingly, Guinea’s pro-poor inequality in health facility delivery and skilled attendance at birth changed to pro-rich inequality, with the reverse being true in the case of use of modern contraceptives for Ghana, Malawi and Rawanda. The good news however is that in a lot of countries, the use of reproductive health services has increased while inequality has decreased within the period under study.

**Conclusion:**

The paper argue that whiles income levels may play a key role in explaining the differences in utilisation and the levels of inequality, indepth studies may be needed to explain the reason for differential improvements and stagnation or deterioration in different countries. In this way, best practices from better performing countries can be documented and adapted by poor performing countries to improve their situation.

## Introduction

The poverty reduction target of the Millennium Development Goals (MDGs) is perhaps the single target achieved by most countries that signed up to the MDGs. The drastic reduction in the percentage of the world’s population that lives in poverty has been attributed to medium to strong growth in incomes in many developing countries [1, 2]. However, growth in average incomes around the world, especially in Low to Middle Income Countries (LMICs) have not necessarily resulted in equitable distribution of incomes [3]. There is evidence to suggest that in developing regions such as Sub-Saharan Africa (SSA), strong economic growth did not result in increased incomes for the population in the poorest quintiles or reduction in inequality [4–6]. For example, SSA’s top 10% income earners at the national level, are responsible for 55% of total income and are second highest to 61% in the Middle East [3]. There are those who have also suggested that SSA belongs to the world’s most unequal regions [7, 8]. The inequality literature also abounds in evidence that suggest that income inequality in SSA is not only higher than the global average, but also higher than inequality in other developing regions of the world [9–12].

Just like the income growth narrative, health outcomes have equally witnessed tremendous improvements over the last two decades. For example, key health outcome indicators such as maternal mortality (987/100,000 in 1990 to 546/100,000 in 2015), neonatal mortality (41.3/1000 in 2000 to 27.7/1000 in 2016), infant mortality (94/1000 in 2000 to 53.3/1000 in 2016) and under-five stunting (43.2% in 2000 to 34.1% in 2016) have all improved drastically over the last 20 years [13, 14]. Access to reproductive health services such as at least four (4+) antenatal visits (43.3% in 2000 to 51.5% in 2014), contraception prevalence among women 15–49 years who are in a union (15.4% in 1994 to 28.4 in 2015) have equally improved substantially in SSA [13, 15]. Estimates from 27 SSA countries with two Demographic and Health Survey (DHS) data points (i.e. 1990 to 2014) suggest that the median health facility births increased from 44 to 57% from the initial to the latest survey [16].

Unlike the income growth and inequality discourse, the health inequality literature is not clear whether improvements in health outcomes in SSA over the last two decades have been equitably distributed and consequently a reduction in health-related inequalities. The existing health inequality literature in SSA [17–22] has mostly been focused on country level rather than comparison of cross-country inequality. Although there are papers that have examined cross-country inequality, they are either focused on developing a service coverage index to monitor the SDGs in general [23–25] or a discussion of methodological considerations for equity oriented monitoring of Universal Health Coverage (UHC). The others [26, 27] used data from a single point to examine health related inequalities. This suggest that there are not many papers that have examined health related inequality over time (using two data points) and across countries. Thus, this paper, using, the use of modern contraceptives, 4+ antenatal visits, health facility deliveries and skilled delivery assistance as proxy measures for use of reproductive health services, examine current levels and changes in utilisation and inequality in utilisation of reproductive health services in SSA, with two rounds of Demographic and Health Surveys (DHS) data. Specifically, the paper:

1. Examine current levels and changes in the use of the four reproductive health services over time in SSA.
2. Examine current levels and changes in inequality in the use of the four reproductive health services over time in SSA.

The rational for using reproductive health services in this paper is based on the fact that reproductive health influences several development outcomes through demographic dividend [28]. For example, adequate and fair access to improved reproductive healthcare lowers fertility rates, sexually transmitted infections (STIs) and improves pregnancy outcomes, with broader individual, family and societal benefits [29]. Such benefits may include a healthier and more productive workforce, access to greater financial and other resources for children, especially those in smaller families [28, 29]. On the contrary, adverse reproductive health outcomes (early and unwanted pregnancies, higher levels of fertility, poorly managed obstetric complications) can limit opportunities for poor women and their families to escape poverty [30, 31]. Additionally, access to adequate and quality reproductive health services is linked to the achievement of several of the SDG 3 targets (e.g. 3.1, 3.2, 3.3 and 3.7). This suggest that adequate and fair access to reproductive health services is key to achieving both the health and non-health related SDGs.

Beside the above, the value of the paper lies in its contribution both to the literature and policy on health-related inequalities. Firstly, the time frame covered by the paper coincide with the time for the implementation of the Millennium Development Goals (MDGs). Thus, the results of the paper can also be interpreted as changes in utilisation and inequality in the use of reproductive health services that occurred during the implementation of the MDGs. The results of the paper can therefore be seen as an evaluation of utilisation and equity implications of changes in reproductive health outcomes during the implementation of the MDGs. The results of the current paper, in addition to prior evidence [23–27] can constitute a good baseline to guide the implementation of SDGs-related health interventions. This is very important, given that the achievement of both the health and non-health related SDGs is to some extent dependent not only on average improvements in access to appropriate reproductive health services, but also, reduction in the levels of inequality in access to reproductive health services. Additionally, the paper is one of the very few in the SSA literature that uses data across several countries to examine health-related inequality at a point in time and over time, making it an important contribution to the health-related inequalities literature. The rest of the paper is made up of four sections. Section 2 covers methods with section 3 and 4 covering results and discussion respectively. Section 5 concludes the paper.

## Methods

### Data

The paper is based on DHS data from 30 SSA countries that have recent DHS data.^1^ Estimates of current levels of utilisation and inequality in the use of reproductive health services is based on DHS data from 30 countries. The second aspect of the study that seeks to estimate changes in the level of use and inequality in the use of reproductive health services is based on DHS data from 21 countries. The reduction is due to the fact that data is not available at two points for 9 countries, hence the reduction to 21 countries.^2^ DHS data is collected via a nationally representative household survey conducted by statistical bureaus of home countries with technical assistance from OR/ICF Macro and ICF International Company. Information collected by DHS surveys in the 30 countries relevant to this study includes use of reproductive health services (use of modern contraceptives, 4+ antenatal visits, health facility deliveries and skilled delivery assistance) and household wealth measured by an asset index. The asset index is calculated using questions on ownership of household assets. The method of principal component analysis is used to derive weights, based on which the first principal component (captures maximum variation in the assets) is calculated, standardised and used as the asset index. The questionnaire used in DHS surveys is based on a model questionnaire developed by the Measure DHS program. Thus, the questionnaire used in each country is principally the same with the exception of a few changes to take care of specific country-level needs. Secondly, questions asked have the same codes and response categories across countries, making it easy to pool the data. For detailed data collection methodology of the DHS see [32–34].

### Variable definition and measurement

The use of modern contraceptives, 4+ antenatal visits, health facility delivery and skilled delivery assistance are used as indicators of reproductive health services. These 4 indicators were selected on the basis that they are key in the package of services under the Safe Motherhood programme [34]. The four indicators are discussed as follows:

#### Antenatal care

Antenatal visits captures the number of antenatal visits made by a pregnant woman (i.e. count form 1,2,3…n). For a long time, WHO has used at least 4 antenatal visits as the benchmark for a pregnant woman to be deemed protected from pregnancy-related risk and complications [35, 36]. Based on this, we assume that any number of antenatal visits fewer than 4 is as risky as not going at all. Thus, the variable is coded as binary; 1 if a woman had 4+ visits, or else 0. Notwithstanding the 4+ benchmark, WHO has recently issued new recommendations that stipulate that the minimum number of antenatal visits should be 8 [37]. Although several reports including that of the DHS continue to use the 4+ visits, a separate variable based on the 8+ is constructed and used in addition to the 4+ antenatal visits. The 8+ antenatal visits indicator is coded as 1 if antenatal visits is 8 or above, else the variable is coded as zero.

#### Use of modern contraceptives

In the survey, women were asked about their current contraceptive use, with the first answer being no use of contraception at all or use of up to 13 other methods of contraception that are either modern or traditional. This variable is recoded into a dummy variable (use of modern contraceptives or not).^3^ Traditionally, contraceptive models have been formulated as use of modern or non-modern methods. This is on the basis that non-modern methods are known to be ineffective and therefore could be likened to a situation of not using contraceptives at all [34]. The variable is coded as 1 if a woman used modern methods and 0 if otherwise. On the basis of this, 2 dummy variables were created for the analysis, one for all women and the other for only women in a union (married or living together).

#### Health facility delivery and skilled delivery assistance

Two dummy variables are respectively used to capture health facility deliveries (i.e. birth occurring in a public or private health facility) and skilled delivery assistance (i.e. deliveries assisted by a doctor, nurse or midwife). The variables are coded 1 if delivery took place in a health facility or was assisted by any of the three health professionals, otherwise the variable is coded 0. The choice of the two variables is on the basis that they give a woman in labour, access to professional delivery services and emergency obstetric care (EOC) where necessary [34]. Although the two variables used may be highly correlated, the two have nonetheless been used together for the purpose of identifying whether there are countries where the two diverge.

### Econometric estimation

To determine the level of inequality in the use of the four reproductive health services, the percentage of the sample in each country using the respective reproductive health service was calculated and grouped according to the lower two and top two quintiles of the chosen socio-economic position variable (asset index) as per Table 1. In addition, the concentration index is used as a measure of socioeconomic inequality in the use of the four reproductive health services, following prior authors [17, 38–40]. The concentration index captures the cumulative proportion of a healthcare variable (in this case reproductive health services) ranked by the individual’s position in a socioeconomic or living standard variable (in this case the asset index) [41]. The concentration index lies between − 1 and 1. A negative value signifies pro-poor distribution of the outcome of interest, with the reverse being true for a positive value of the concentration index.

**Table 1:** Use of Reproductive Health Services in Sub-Saharan Africa by Wealth/Asset Quintiles

Countries	Health Facility Delivery		Skilled Delivery Assistance		4+ Antenatal Visits		Use of Modern Contraception	
	Quintile 1 & 2	Quintile 4 & 5	Quintile 1 & 2	Quintile 4 & 5	Quintile 1 & 2	Quintile 4 & 5	Quintile 1 & 2	Quintile 4 & 5
Angola	23.89	44.85	27.03	42.28	35.21	36.87	8.56	70.42
Benin	38.71	39.69	36.78	41.55	33.11	45.1	26.89	52.36
Burkina-Faso	30.89	47.59	20.75	64.18	30.62	47.9	17.78	69.8
Burundi	33.88	48.79	33.85	48.94	39.55	43.36	26.12	57.71
Cameroon	24.93	50.89	25.29	50.19	30.82	47.52	14.47	67.1
Chad	25.28	62.69	24.06	64.88	30.94	51.74	23.21	63.78
CID	30.83	47.43	30.9	47.19	30.13	48.72	23.4	57.66
Comoros	38.06	40.02	38.61	39.89	39.25	39.94	30.18	49.71
Congo	65.89	20.69	66.22	20.51	64.3	22.34	47.16	36.62
DRC	40.88	38.22	42.29	37.11	40.65	40.06	21.36	64.95
Ethiopia	25.83	62.13	26.37	62.46	29.95	56.75	23.7	60.88
Gabon	61.31	22.3	61.14	22.46	58.09	25.36	50.21	32.31
Gambia	39.86	42.76	40.57	42.04	47.62	31.71	27.93	58.41
Ghana	41.25	38.24	41.37	38.08	48.52	32.37	44.69	33.12
Guinea	22.66	59.55	17.52	66.33	31.65	48.47	12.94	76.16
Kenya	33.41	46.82	33.93	46.34	42.30	39.46	33.49	43.61
Lesotho	36.41	41.39	37.11	40.97	40.96	38.74	32.41	47.4
Liberia	50.35	26.19	49.75	26.71	54.19	22.67	43.66	32.46
Malawi	41.26	39.71	41.21	39.74	39.83	41.35	35.73	44.97
Mali	22.87	61.19	17.27	71.50	23.36	60.9	13.84	76.68
Namibia	36.94	40.3	59.14	49.96	38.50	39.78	30.21	48.28
Niger	15.57	72.68	14.82	73.61	26.23	57.59	10.88	79.94
Nigeria	15.37	64.05	14.26	65.23	24.13	53.19	8.95	72.18
Rwanda	42.51	38.56	42.53	38.53	42.49	37.96	38.36	42.32
Senegal	46.09	29.17	43.57	32.09	46.77	30.13	41.53	33.12
Sierra Leone	35.69	46.53	30.39	53.3	39.85	40.44	22.03	65.06
Tanzania	29.16	52.2	28.4	53.17	32.42	49.49	27.72	52.44
Togo	35.45	43.63	16.18	67.49	39.35	43.06	36.86	46.6
Zambia	36.13	41.2	34.28	42.99	42.62	34.6	30.88	45.45
Zimbabwe	30.41	54.27	30.59	54.07	34.34	49.54	29.9	55
West Africa	33.37	46.14	29.98	50.71	36.46	42.73	25.96	56.15
East and Central Afr.	38.11	42.56	38.6	42.15	41.03	40.09	30.74	49.76
Southern Africa	37.71	42.14	37.43	42.48	39.47	40.66	32.6	47.57
Sub-Saharan Africa	35.99	44.02	35.04	45.5	38.61	41.43	29.96	50.9

Source: Constructed by Author Based on DHS data. Note that quintile 1 & 2 are the 2 lowest quintiles in the household asset distribution and quintile 4 & 5 are the 2 topmost quintiles

Assuming *h* is a reproductive health services indicator (use of modern contraceptives, 4+ and 8+ antenatal visits, health facility deliveries and skilled delivery assistance) for a woman *i*, the concentration index (*CI*) can be calculated using Eq. 1 below for individual level data as in the current case.

1 $$ CI=\frac{2}{N\mu}\sum \limits_{i=1}^N{w}_i{h}_i{r}_i-1 $$

Where

2 $$ \mu =\frac{1}{N}\sum \limits_{i=1}^N{w}_i{h}_i $$

is the weighted mean of the health variable in the sample, *N* is the sample size, *w*_*i*_ is the sample weight, where the sum of *w*_*i*_ is equal to *N* and *r*_*i*_ is the fractional rank of the *i*th individual in the living standard’s distribution. Given that income or expenditure is not available in the DHS data, an assets index is used as the living standard measure. For weighted data, *r*_*i*_ can be defined as in Eq. 3, where *w*_0_ = 0

3 $$ {r}_i=\frac{1}{N}\sum \limits_{j=1}^{i=1}{w}_j+\frac{1}{2}{w}_i $$

Additionally, the paper also examines changes in inequality overtime using two data points as already indicated. To do that, concentration indices for the two data points are calculated, followed by the ratio of the earlier concentration index (t-1) to the later concentration index (t), to determine the extent to which the concentration index has changed between the two periods.

The Erreyger’s analogue of the standard concentration index was also calculated (results not shown but available on demand) to overcome the problem of the bounds of the concentration index not lying between − 1 and 1 for a dichotomous variable [42, 43]. It is important to emphasise that the Wagstaff normalisation which has also been mentioned in the literature as an alternative solution could have been used, but was not chosen given that it has equally been criticised by Erreygers [43]. All calculations were carried out using the healthcare module of ADePT Version 5.4. In addition, STATA Version 13 was also used to calculate frequencies and also to confirm the concentration indices calculated with the ADePT software.

## Findings

### Current levels and changes in the use of reproductive health services

The results in Table 2 shows that on the average, health facility deliveries, skilled delivery assistance and 4+ antenatal visits in SSA are 63.3, 58.9 and 53.8% respectively. At the sub-regional level, Southern Africa has the highest utilisation rate for all the four reproductive health services followed by East and Central Africa and West Africa. It is important though to note that in the case of 4+ antenatal visits, West Africa performed better than East and Central Africa. At the country level, Benin, Congo, Gabon, Malawi, Namibia, Rwanda and Zimbabwe have over 80% utilisation of health facility deliveries, skilled delivery assistance and 4+ antenatal visits, with Chad, Ethiopia, Nigeria, Niger and Burundi having less than 40% utilisation of some or all the three reproductive health services mentioned. In the case of modern contraceptives, utilisation rates are generally low with the SSA average being 19.7%. At the individual country level, it is only Namibia that has approximately 50%, followed by Zimbabwe (48.1%), Lesotho (48.2%), Malawi (44.7%), Kenya (35.4%) and Zambia (31.4%). The remaining countries have modern contraception utilisation rate less than 30%, with the lowest being 3% for Chad.

**Table 2:** Use of Reproductive Health Services in Sub-Saharan Africa

Countries	Health Facility Delivery		Skilled Delivery Assistance		4+ Antenatal Visits		Use of Modern Contraceptives	
	No	Yes	No	Yes	No	Yes	No	Yes
Angola	54.0	46.0	49.6	50.4	42.4	57.6	90.7	9.3
Benin	12.9	87.1	19.1	80.9	40.6	59.4	91.5	8.5
Burkina-Faso	26.2	73.8	74.7	25.4	65.4	34.6	85.3	14.8
Burundi	34.6	65.4	33.9	66.1	66.2	33.8	88.5	11.5
Cameroun	34.0	66.0	31.4	68.6	37.0	63.0	84.0	16.0
Chad	79.4	20.6	80.2	19.8	71.5	28.5	97.0	3.0
CIV	42.6	57.4	40.9	59.1	56.6	43.4	86.8	13.2
Comoros	21.6	78.4	15.1	84.9	41.1	58.9	90.5	9.5
Congo	13.6	86.4	12.4	87.6	26.2	73.8	81.8	18.2
DRC	24.8	75.2	21.0	79.0	54.7	45.4	93.1	6.9
Ethiopia	62.5	37.5	62.8	37.2	63.7	36.3	79.6	20.4
Gabon	15.1	84.9	16.5	83.5	30.8	69.2	80.6	19.4
Gambia	39.8	60.2	38.3	61.7	21.7	78.3	93.8	6.2
Ghana	28.0	72.0	27.2	72.9	13.5	86.5	81.5	18.5
Guinea	59.3	40.7	57.8	42.2	43.2	56.8	94.1	5.9
Kenya	41.4	58.6	40.5	59.5	45.7	54.3	64.6	35.4
Lesotho	23.5	76.5	22.0	78.0	25.2	74.8	51.8	48.2
Liberia	44.0	56.0	40.2	59.8	23.9	76.1	80.5	19.6
Malawi	6.9	93.1	8.4	91.6	49.0	51.0	55.3	44.7
Mali	41.3	58.7	57.2	42.8	57.2	42.8	89.9	10.1
Namibia	12.8	87.3	11.8	88.2	19.8	80.2	50.1	49.9
Niger	60.0	40.0	60.4	39.6	66.8	33.2	91.0	9.0
Nigeria	61.7	38.3	59.9	40.1	46.5	53.5	88.6	11.4
Rwanda	8.8	91.2	8.8	91.2	55.9	44.1	72.6	27.4
Senegal	28.3	71.7	47.2	52.8	51.6	48.4	85.2	14.8
Sierra-Leone	41.4	58.6	49.9	50.1	12.5	87.5	79.2	20.8
Tanzania	34.3	65.7	37.1	62.9	50.3	49.7	75.8	24.1
Togo	29.1	70.9	61.0	39.0	44.5	55.5	83.2	16.8
Zambia	27.6	72.4	31.6	68.4	45.1	54.9	68.6	31.4
Zimbabwe	17.7	82.3	16.7	83.3	23.6	76.4	51.9	48.1
West Africa	40.8	59.2	50.9	49.1	44.8	55.2	87.0	13.0
East & Cent. Afr.	40.0	60.0	39.0	61.0	50.5	49.5	81.4	18.6
Southern. Africa	16.3	83.7	17.1	82.8	39.7	60.3	59.5	40.5
SSA	36.7	63.3	41.1	58.9	46.2	53.8	80.3	19.7

Source: Author’s calculation based on DHS Data

In addition to current levels of utilisation, the results in Table 3 shows changes in utilisation of the four reproductive health services for selected countries over a period of not less than 10 years. The change is simply the difference between utilisation at the first data point (*t-1)* less utilisation at the current data point (*t).* Thus, the change is not interpreted as change in terms of percentage points but change in percentage utilisation. The results suggest that on the average, utilisation of the four reproductive health services have increased by 6.8% (4+ antenatal visits) and 17.2% (health facility delivery). At the sub-regional level, the highest rate of change occurred in Southern Africa, followed by East and Central Africa and West Africa. However, in the case of 4+ antenatal visits, utilisation dropped by 6.7% in Southern Africa. At the individual country level, the results show that the three countries with the largest improvements were (1) Rwanda (60.5%) Burkina Faso (35.7%) and Malawi (35.8%) for health facility deliveries; (2) Rwanda (60.2%), Malawi (34.5%) and Ghana (28.3%) for skilled delivery assistance, (3) Rwanda (30.8%), Niger (19%) and Ghana (15.9%) for 4+ antenatal visits; (4) Malawi (23%), Lesotho (21.4%) and Rwanda (21.9%) for use of modern contraceptives. However, the lowest increase or decrease was recorded by (1) Gabon (2.3%), Chad (− 2.7%), Cote de’ Ivoire (− 3.4%) for health facility delivery, (2) Togo (− 9.2%), Chad (− 8.3%), and Burkina Faso (− 7.8) for skilled delivery assistance, (3) Zambia (− 17.9), Malawi (− 6.7%) and Benin (− 2.7) for 4+ antenatal visits and (4) Chad (0.4%), Cote de’ Ivoire (0.7%) and Guinea (0.8%) for use of modern contraceptives.

**Table 3:** Trends in the Use of Reproductive Health Services Over time in Sub-Saharan

Countries	Health Facility Delivery			Skilled Delivery Assistance			4+ Antenatal Visits			Use of Modern Contraceptives		
	t-1%	t %	Δ %	t-1%	t %	Δ %	t-1%	t %	Δ %	t-1%	t %	Δ %
Benin (2001–2012)	77.3	87.1	9.8	74.5	80.9	6.4	62.1	59.4	−2.7	6.6	8.5	1.9
Burkina-Faso (98–2010)	38.1	73.8	35.7	33.2	25.4	−7.8	24.6	34.6	10.0	7.5	14.8	7.4
Cameroon (1998–2011)	60.0	66.0	6.0	64.1	68.6	4.6	57.0	63.0	6.0	9.4	16.0	6.6
Chad (2004–2015)	23.3	20.6	−2.7	28.1	19.8	−8.3	25.4	28.5	3.1	2.7	3.0	0.4
CIV (1998–2012)	60.8	57.4	−3.4	61.1	59.1	−2.0	44.7	43.4	−1.3	12.5	13.2	0.7
Ethiopia (2005–2016)	12.3	37.5	25.2	12.7	37.2	24.5	17.3	36.3	19.0	10.9	20.4	9.5
Gabon (2000–2012)	82.6	84.9	2.3	78.5	83.5	5.0	58.5	69.2	10.7	12.1	19.4	7.3
Ghana (2003–2014)	43.5	72.0	28.5	44.6	72.9	28.3	70.6	86.5	15.9	14.6	18.5	3.9
Guinea (1999–2012)	31.4	40.7	9.3	38.0	42.2	4.2	51.7	56.8	5.1	5.1	5.9	0.8
Kenya (2003–2014)	44.1	58.6	14.5	45.5	59.5	14.0	53.5	54.3	0.8	22.6	35.4	12.8
Lesotho (2004–2014)	55.2	76.5	21.3	58.2	78.0	19.8	71.5	74.8	3.3	26.8	48.2	21.4
Malawi (2004–2016)	57.4	93.1	35.8	57.1	91.6	34.5	57.7	51.0	−6.7	21.7	44.7	23.0
Mali (2001–2013)	37.0	58.7	21.7	40.0	42.8	2.8	29.6	42.8	13.2	5.7	10.1	4.4
Namibia (2000–2013)	75.9	87.3	11.4	77.2	88.2	11.0	74.5	80.2	5.8	40.9	49.9	9.0
Niger (1998–2012)	24.1	40.0	15.9	23.7	39.6	15.9	14.2	33.2	19.0	5.7	9.0	3.3
Nigeria (2003–2013)	37.1	38.3	1.2	41.1	40.1	−1.0	51.1	53.5	2.4	8.2	11.4	3.2
Rwanda (2005–2015)	30.7	91.2	60.5	31.0	91.2	60.2	13.3	44.1	30.8	5.5	27.4	21.9
Senegal (2005–2016)	61.2	71.7	10.5	51.0	52.8	1.8	39.5	48.4	8.9	7.0	14.8	7.8
Togo (1998–2014)	47.4	70.9	23.5	48.2	39.0	−9.2	47.2	55.5	8.3	7.9	16.8	8.9
Zambia (2002–2014)	43.2	72.4	29.2	43.0	68.4	25.4	72.8	54.9	−17.9	17.3	31.4	14.1
Zimbabwe (1999–2015)	75.6	82.3	6.7	73.5	83.3	9.8	75.0	76.4	1.4	33.9	48.1	14.2
West Africa	44.9	59.4	14.5	40.6	47.6	7	40.7	49.7	9	7.5	12.2	4.7
East & Cent, Africa	35.4	54.4	19	36.5	54.7	18.2	31.6	47.5	15.9	10.6	22.1	11.5
Southern Africa	59.1	84	24.9	59.1	82.7	23.6	67.1	60.4	−6.7	26.6	43	16.4
Sub-Saharan Africa	45.6	62.8	17.2	43.8	56.7	12.9	44.3	51.1	6.8	12.8	21.6	8.8

Source: Author’s calculation based on DHS Data. Note that *t-1* corresponds to the first date in the parentheses and *t* the second date

On the contrary, the results (See Tables 4 and 5) are a bit different when 8+ antenatal visits and utilisation of modern conception restricted to women in union are used, compared to 4+ antenatal visits and use of modern contraception by all women. As expected, the percentage of women making 8+ antenatal visits are much lower in all countries compared to 4+ antenatal visits. At the sub-regional level for example, the use of the 8+ indicator reduced antenatal use from 55.2 to 9.7% for West Africa, 49.5 to 3.3% for East and Central Africa and 60.3 to 5.9% for Southern Africa. At the individual country level, the difference in utilisation between the two antenatal indicators are as high as 92% (Malawi), 83%(Congo), 78%(Gabon) and as low as 13%(Nigeria), 16% (Sierra Leone) and 20(Chad). In the case of modern contraception restricted to women in union compared to all women, sub-regional utilisation remained almost the same for West Africa (13% to 13.08) but changed from 18.6 to 27.7% for East and Central Africa and 40.5 to 55.4% for Southern Africa. At the individual country level, rates tend to increase when use of modern contraception for only women in union is used, except for countries such as Nigeria, Gabon, Liberia, Ethiopia, Guinea and Congo. For example, with the use of modern contraception for women in union, utilisation rates increased by 20% (Rwanda), 18% (Malawi) and 14% (Malawi).

**Table 4:** Trends in Utilisation and Inequality in the Use of Antenatal care and Modern Contraception

Country	8+ Antenatal Visit						Use of Modern Contraception for Women in Union					
	Yes	Quantile		Concentration Index			Yes	Quantile		Concentration Index		
	%	1 & 2	4 & 5	Rank	CI	*p*-value	%	1 & 2	4 & 5	Rank	CI	*p*-value
Angola	6.23	26.31	48.09	13	0.3382	0.000	8.86	9.55	73.25	30	0.5002	0.000
Benin	10.23	17.04	68.72	20	0.4577	0.000	7.37	28.80	49.14	18	0.1984	0.000
Burkina-Faso	0.31	3.13	87.50	29	0.7484	0.000	15.48	19.83	66.38	23	0.3360	0.000
Burundi	0.41	15.00	80.00	25	0.5117	0.000	18.42	27.25	55.83	14	0.1189	0.000
Cameroun	7.81	13.85	69.60	19	0.4431	0.000	14.20	15.87	64.44	25	0.3527	0.000
Chad	0.90	24.49	66.33	17	0.3773	0.000	3.03	24.81	58.97	21	0.2591	0.000
CIV	3.12	13.69	76.78	27	0.6033	0.000	11.41	31.93	49.46	20	0.2241	0.000
Comoros	13.53	28.21	49.57	6	0.1903	0.000	13.55	30.50	50.45	7	0.0674	0.012
Congo	3.44	44.34	43.43	14	0.3413	0.000	16.12	50.00	33.82	17	0.1862	0.000
DRC	2.59	47.42	36.08	1	0.0371	0.175	6.35	23.16	62.79	24	0.3438	0.000
Ethiopia	3.55	11.77	84.31	28	0.6166	0.000	29.38	44.85	41.32	15	0.1526	0.000
Gabon	6.77	36.10	46.20	12	0.3036	0.000	15.56	48.58	32.21	10	0.0893	0.000
Gambia	4.74	40.00	43.92	7	0.2067	0.000	7.49	29.40	55.90	22	0.2760	0.000
Ghana	28.84	32.23	47.16	10	0.2531	0.000	22.51	48.69	30.94	2	−0.0254	0.082
Guinea	8.86	17.01	69.85	18	0.4417	0.000	3.35	18.06	66.96	26	0.3551	0.000
Kenya	2.71	23.02	62.63	23	0.4902	0.000	47.18	33.46	43.77	9	0.0888	0.000
Lesotho	11.80	26.98	53.62	11	0.2999	0.000	59.27	35.20	44.93	5	0.0487	0.000
Liberia	21.92	50.63	27.84	4	0.1334	0.000	18.62	50.45	25.32	11	0.1096	0.000
Malawi	1.40	40.11	39.58	2	0.0749	0.121	57.89	35.10	45.06	1	0.0215	0.000
Mali	4.98	16.31	70.70	15	0.3548	0.000	10.46	15.21	74.51	27	0.3948	0.000
Namibia	25.91	29.26	49.75	5	0.1728	0.000	55.44	30.93	48.40	8	0.0691	0.000
Niger	0.20	6.67	53.33	8	0.2163	0.037	10.28	11.15	79.55	28	0.4234	0.000
Nigeria	25.78	12.06	69.28	21	0.4728	0.000	9.47	10.22	71.93	29	0.4793	0.000
Rwanda	0.07	0.00	100.00	30	0.9193	0.000	47.45	37.11	42.95	3	0.0258	0.000
Senegal	0.77	42.85	44.76	16	0.3730	0.000	19.31	40.99	33.94	16	0.1720	0.000
Sierra-Leone	42.73	36.32	45.21	3	0.0818	0.000	15.09	27.92	56.75	19	0.2109	0.000
Tanzania	0.91	14.06	75.00	26	0.5245	0.000	29.00	28.97	50.66	12	0.1097	0.000
Togo	2.72	22.05	70.59	22	0.4856	0.000	17.23	44.43	37.40	6	0.0591	0.000
Zambia	0.86	22.50	58.75	24	0.4998	0.000	43.10	31.67	45.40	13	0.1186	0.000
Zimbabwe	12.34	22.01	64.54	9	0.2441	0.000	66.12	31.30	53.58	4	0.0373	0.000
West Africa	9.76	16.50	65.97		0.5865	0.000	13.08	27.93	53.83		0.2404	0.000
East & Central Africa	3.22	20.18	66.07		0.5520	0.000	27.73	31.71	47.70		0.2108	0.000
Southern Africa	5.96	27.48	54.20		0.4144	0.000	55.39	33.37	46.99		0.0686	0.000
Sub-Saharan Africa	7.07	18.80	64.06		0.5475	0.000	25.62	31.42	48.97		0.1067	0.000

Source: Author’s calculation based on DHS data

**Table 5:** Trends in Utilisation and Inequality in the Use of Antenatal care and Modern Contraception

Country	8+ Antenatal Visit						Modern Contraception for Women in Union					
	Percentages			Concentration Index			Percentages			Concentration Index		
	t-1	t	% ∆	t-1	t	Times ∆	t-1	t	%	t-1	t	Times ∆
Benin (2001–2012)	15.1	10.2	−4.9	0.1758	0.4577	1.6	6.7	7.4	0.7	0.0624	0.1984	2.2
Burkina- (1998–2010)	0.6	0.3	−0.3	0.3091	0.7484	1.4	6.2	15.5	9.3	0.2604	0.3360	0.3
Cameroon (1998–2011)	8.4	7.8	−0.6	0.1240	0.4431	2.6	8.3	14.2	5.9	0.2231	0.3527	0.6
Chad (2004–2015)	1.7	0.9	−0.8	0.4708	0.3773	−0.2	2.9	3.0	0.1	0.7341	0.2591	−0.6
CIV (1998–2012)	2.8	3.1	0.3	0.0477	0.6033	11.6	9.7	11.4	1.7	0.0514	0.2241	3.4
Ethiopia (2005–2016)	3.7	3.6	− 0.1	0.8327	0.6166	−0.3	16.0	29.4	13.4	0.4019	0.1526	−0.6
Gabon (2000–2012)	6.1	6.8	0.7	0.2108	0.3036	0.4	10.2	15.6	5.4	0.1213	0.0893	−0.3
Ghana (2003–2014)	22.0	28.8	6.8	0.3364	0.2531	−0.2	17.5	22.5	5	0.1817	−0.0254	−1.1
Guinea (1999–2012)	10.2	8.9	−1.3	0.0159	0.4417	26.8	4.4	3.4	−1	0.0177	0.3551	19.0
Kenya (2003–2014)	10.0	2.7	−7.3	0.2658	0.4902	0.8	31.4	47.2	15.8	0.2311	0.0888	−0.6
Lesotho (2004–2014)	17.5	11.8	−5.7	0.2776	0.2999	0.1	33.9	59.3	25.4	0.2225	0.0487	−0.8
Malawi (2004–2016)	5.0	1.4	−3.6	0.1635	0.0749	−0.5	26.8	57.9	31.1	0.1156	0.0215	−0.8
Mali (2001–2013)	4.1	5.0	0.9	0.3819	0.3548	−0.1	5.5	10.5	5	0.2637	0.3948	0.5
Namibia (2000–2013)	22.5	25.9	3.4	0.1383	0.1728	0.2	46.1	55.4	9.3	0.1160	0.0691	−0.4
Niger (1998–2012)	0.1	0.2	0.1	0.0000	0.2163	...	6.2	10.3	4.1	0.1070	0.4234	3.0
Nigeria (2003–2013)	26.2	25.8	−0.4	0.4075	0.4728	0.2	7.4	9.5	2.1	0.4174	0.4793	0.1
Rwanda (2005–2015)	0.2	0.1	−0.1	0.7859	0.9193	0.2	10.0	19.3	9.3	0.3001	0.0258	−0.9
Senegal (2005–2016)	0.6	0.8	0.2	0.5634	0.3730	−0.3	9.3	19.3	10	0.3943	0.1720	−0.6
Togo (1998–2014)	4.2	2.7	−1.5	0.1473	0.4856	2.3	6.9	17.2	10.3	0.0519	0.0591	0.1
Zambia (2002–2014)	12.8	0.9	−11.9	−0.0111	0.4998	−45.9	20.5	43.1	22.6	0.0054	0.1186	20.9
Zimbabwe (1999–2015)	15.3	12.3	−3	0.1172	0.2441	1.1	47.9	66.1	18.2	0.0734	0.0373	−0.5
West Africa	7.35	9.76	2.41	0.2669	0.5865	1.2	8.6	13.1	4.5	0.4123	0.2404	−0.4
East & Cent Africa	4.27	3.22	−1.05	0.4295	0.5220	0.2	15.3	27.7	12.4	0.2849	0.2108	−0.3
Southern Africa	12.25	5.96	−6.29	0.3744	0.4144	0.1	29.0	55.4	26.4	0.1589	0.0686	−0.6
SSA	7.61	7.07	−0.54	0.5475	0.5475	0.7	16.6	25.6	9	0.1399	0.1067	−0.2

Source: Author’s calculation based on DHS data

### Inequality in the use of reproductive health services

In this section we present results on how the use of the four reproductive health services are distributed within the population. First, we compare utilisation of the four reproductive health services between the lowest two (poorest and poorer) and top two (richer and richest) quintiles of household assets as in Table 1. At the SSA level, percentage utilisation of all the four reproductive health services is higher for the top two quintiles compared to the lowest two. For instance, utilisation rates were 1.2 times (health facility deliveries), 1.3 times (skilled delivery assistance), 1.1 times (4+ antenatal visits) and 1.7 times (use of modern contraception) higher in the top two asset quintiles compared to the lowest two asset quintiles. At the sub-regional level, West Africa has the most unequitable distribution of use of the four reproductive health services, as utilisation rates are higher in the top two asset quintiles compared to the lowest two. This is followed by Southern Africa in the case of skilled delivery assistance and 4+ antenatal visits. The difference in the utilisation of health facilities for deliveries between the poor and the rich is the same but higher in East and Central Africa for use of modern contraceptives than in Southern Africa. At the individual country level, the rich compared to the poor, have a substantially higher utilisation of (1) health facilities for delivery in Nigeria and Niger (2) skilled delivery assistance in Nigeria, Niger, Burkina Faso and Togo, (3) 4+ antenatal visits in Niger, Nigeria and Mali and (4) use of modern contraceptives in Niger, Nigeria, Mali, Cameroon and Guinea. On the other hand, countries like Congo, Gabon and Liberia have the least difference in utilisation rates between the poor and the rich.

To confirm the results in Table 1, concentration indices were computed for the use of each of the four reproductive health services across all the countries in the study sample. The results confirm the earlier results that the use of the four reproductive health services is concentrated among the rich in SSA and that inequality in the use of the four reproductive health services is highest in West Africa followed by East and Central Africa and then Southern Africa. At the individual country level, the results are not entirely different. Use of all the four reproductive health services are concentrated among the rich with the exception of use of modern contraceptives that is concentrated among the poor in Rwanda, Malawi, Ghana and Zimbabwe. The results equally confirm that countries such as Angola, Ethiopia, Niger, Nigeria, Guinea, Mali, Cote de’ Ivoire and Togo have some of the most unequitable distribution of the use of reproductive health services in SSA, with the *p*-values suggesting that the estimates are significant. The Erreyger’s standardization (results not shown but available on demand) was equally used to compute the concentration index. The results confirm the results in Table 6, except that in the Erreyger’s standardization, additional countries; Burkina Faso (use of modern contraceptives), Cameroon (health facility deliveries, skilled delivery assistance and 4+ antenatal visits) and Sierra Leone (use of modern contraceptives) had higher levels of inequality in the use of reproductive health services.

**Table 6:** Concentration Indices and P-values for use of Reproductive Health Services in Sub-Saharan Africa – Standard Concentration Indices

Countries	Health Fac. Deliveries			Delivery Assistance			4+ Antenatal Visit			Modern Contraception		
	Rank	CI	*p*-value	Rank	CI	*p*-value	Rank	CI	*p*-value	Rank	CI	*p*-value
Angola	27	0.3150	0.000	22	0.2804	0.000	26	0.1930	0.000	30	0.4315	0.000
Benin	6	0.0669	0.000	11	0.0954	0.000	21	0.1536	0.000	18	0.1700	0.000
Burkina-Faso	13	0.1175	0.000	26	0.3448	0.000	19	0.1345	0.000	25	0.3165	0.000
Burundi	9	0.0812	0.000	8	0.0820	0.000	1	0.0018	0.443	16	0.1168	0.000
Cameroun	24	0.2450	0.000	21	0.2349	0.000	25	0.1721	0.000	24	0.3072	0.000
Chad	25	0.2957	0.000	25	0.3394	0.000	22	0.1542	0.000	23	0.2947	0.000
CIV	22	0.2185	0.000	20	0.2133	0.000	29	0.2375	0.000	19	0.1938	0.000
Comoros	8	0.0786	0.000	7	0.0703	0.000	14	0.0945	0.000	6	0.0455	0.000
Congo	4	0.0472	0.000	4	0.0419	0.000	12	0.0757	0.000	17	0.1688	0.000
DRC	10	0.0843	0.000	6	0.0668	0.000	15	0.1082	0.000	22	0.2857	0.000
Ethiopia	29	0.3185	0.000	23	0.3224	0.000	27	0.2218	0.000	11	0.0718	0.000
Gabon	3	0.0420	0.000	3	0.0411	0.000	13	0.0796	0.000	15	0.1125	0.000
Gambia	15	0.1301	0.000	12	0.1232	0.000	2	0.0170	0.001	20	0.2148	0.000
Ghana	18	0.1416	0.000	13	0.1394	0.000	9	0.0504	0.000	1	−0.0435	0.002
Guinea	26	0.3017	0.000	28	0.3658	0.000	24	0.1612	0.000	28	0.4044	0.000
Kenya	21	0.1920	0.000	18	0.1879	0.000	16	0.1152	0.000	10	0.0626	0.000
Lesotho	12	0.0927	0.000	10	0.0875	0.000	10	0.0576	0.000	5	0.0272	0.001
Liberia	16	0.1334	0.000	14	0.1414	0.000	11	0.0579	0.000	14	0.1052	0.000
Malawi	1	0.0177	0.000	1	0.0182	0.000	8	0.0413	0.000	4	−0.0078	0.070
Mali	23	0.2319	0.000	27	0.3547	0.000	28	0.2344	0.000	26	0.3617	0.000
Namibia	5	0.0531	0.000	5	0.0504	0.000	5	0.0308	0.000	8	0.0465	0.000
Niger	28	0.3158	0.000	24	0.3303	0.000	17	0.1205	0.000	27	0.3849	0.000
Nigeria	30	0.4163	0.000	30	0.4231	0.000	30	0.2945	0.000	29	0.4062	0.000
Rwanda	2	0.0289	0.000	2	0.0286	0.000	4	0.0188	0.016	2	−0.0220	0.004
Senegal	17	0.1388	0.000	19	0.1925	0.000	23	0.1611	0.000	12	0.0780	0.000
Sierra-Leone	7	0.0765	0.000	16	0.1700	0.000	3	0.0176	0.000	21	0.2164	0.000
Tanzania	20	0.1674	0.000	17	0.1745	0.000	18	0.1206	0.000	9	0.0585	0.000
Togo	19	0.1533	0.000	29	0.3759	0.000	20	0.1345	0.000	13	0.0815	0.000
Zambia	14	0.1218	0.000	15	0.1462	0.000	7	0.0363	0.000	7	0.0462	0.000
Zimbabwe	11	0.0850	0.000	9	0.0828	0.000	6	0.0311	0.000	3	−0.0097	0.082
West Africa		0.4126	0.000		0.2515	0.000		0.1621	0.000		0.1437	0.000
East & Cen. Afri.		0.1127	0.000		0.1061	0.000		0.1508	0.000		0.2070	0.000
Southern Africa		0.0623	0.000		0.0744	0.000		0.0632	0.000		0.0011	0.364
SSA		0.1007	0.000		0.1405	0.000		0.1345	0.000		0.0824	0.000

Source: Author’s Calculation based on DHS data

As earlier indicated, 8+ antenatal visits and use of modern contraceptives by women in union was also used (see results in Tables 4 and 5). At the sub-regional level, using the 8+ indicator increased the difference in utilisation between women in the lower two and top two quintiles substantially from − 0.9 to 49% for west Africa, 6.3 to 45.9% for East and Central Africa, 1.2 to 26.7% for Southern Africa and 2.8 to 45.3% for SSA. At the individual country level, the results suggest higher levels of inequality compared to the using 4+ antenatal visits. For example, differences in antenatal visits between women in the lower two and top two quintiles increased substantially in the case of 8+ antenatal visits for Burkina Faso (17.3 to 84.3%), Burundi (3.8 to 65%), Rwanda (− 4.5 to 100%), Togo (3.7 to 48.5%) and Zambia (− 8 to 36%). The trend is the same for the concentration index, with Burkina Faso, Burundi, Rwanda, Zambia and Togo having the highest difference at the country level, whiles West Africa tops the sub-regional level followed by East and Central Africa and Southern Africa. On the contrary, restricting the use of modern contraceptives to women in union reduces pro-rich inequalities with the exception of a few countries such as Rwanda and Angola, where pro-rich inequality increased marginally. For example, a pro-rich difference of 43.3% reduced to 17.5% for CIV, 63 to 49% for Guinea, a pro-rich difference of 37% to pro-poor difference of 3.5% for Ethiopia, a pro-rich difference 7.9% to a pro-poor difference of 7% for Togo and a pro-poor difference of 11.2 to 25% for Liberia, with the trend being the same for the concentration indices.

### Changes in inequality in the use of reproductive health services

In this section we present results on changes in inequality in the use of the four reproductive health services over a period of not less than 10 years as in Table 7. It is important to state that the cross-country differences in the time of collecting the two datasets can to a certain extent affect cross-country changes in inequality in the use of the four reproductive health services. Given however, that the DHS does not have data collected at the same time for all the countries, the results remains valid.

**Table 7:** Changes in Inequality in the Use of Reproductive Health Services in Sub-Saharan Africa – Standard Concentration Indices

Countries	Health Facility Delivery			Skilled Delivery Assistance			4+ Antenatal Visits			Use of Mod. Contraceptives		
	t-1	t	Times Δ	t-1	t	Times Δ	t-1	t	Times Δ	t-1	t	Times Δ
Benin (2001–2012)	0.0492	0.0669	1.36	0.0527	0.0954	1.81	0.0431	0.1536	3.56	0.0777	0.17	2.19
Burkina-Faso (1998–2010)	0.1856	0.1175	0.63	0.2053	0.3448	1.68	0.0412	0.1345	3.26	0.2700	0.3165	1.17
Cameroon (1998–2011)	0.1580	0.2450	1.55	0.1524	0.2349	1.54	0.1194	0.1721	1.44	0.1832	0.3072	1.68
Chad (2004–2015)	0.5566	0.2957	0.53	0.5392	0.3394	0.63	0.4214	0.1542	0.37	0.7068	0.2947	0.42
CIV (1998–2012)	0.0568	0.2185	3.85	0.0533	0.2133	4.00	0.0305	0.2375	7.79	0.0601	0.1938	3.22
Ethiopia (2005–2016)	0.7090	0.3185	0.45	0.7103	0.3224	0.45	0.4592	0.2218	0.48	0.3201	0.0718	0.22
Gabon (2000–2012)	0.0456	0.0420	0.92	0.0576	0.0411	0.71	0.0822	0.0796	0.97	0.1246	0.1125	0.90
Ghana (2003–2014)	0.3213	0.1416	0.44	0.3092	0.1394	0.45	0.1063	0.0504	0.47	0.1215	− 0.0435	−0.36
Guinea (1999–2012)	−0.0277	0.3017	−10.89	− 0.0315	0.3658	−11.61	0.0230	0.1612	7.01	0.0356	0.4044	11.36
Kenya (2003–2014)	0.2899	0.1920	0.66	0.2832	0.1879	0.66	0.1130	0.1152	1.02	0.1877	0.0626	0.33
Lesotho (2004–2014)	0.1877	0.0927	0.49	0.1791	0.0875	0.49	0.0763	0.0576	0.75	0.2032	0.0272	0.13
Malawi (2004–2016)	0.1324	0.0177	0.13	0.1333	0.0182	0.14	0.0578	0.0413	0.71	0.0960	−0.0078	−0.08
Mali (2001–2013)	0.1338	0.2319	1.73	0.2368	0.3547	1.50	0.1249	0.2344	1.88	0.2647	0.3617	1.37
Namibia (2000–2013)	0.0534	0.0531	0.99	0.0532	0.0504	0.95	0.0412	0.0308	0.75	0.0951	0.0465	0.49
Niger (1998–2012)	0.1147	0.3158	2.75	0.1193	0.3303	2.77	0.0359	0.1205	3.36	0.0913	0.3849	4.22
Nigeria (2003–2013)	0.3631	0.4163	1.15	0.3459	0.4231	1.22	0.2827	0.2945	1.04	0.3576	0.4062	1.14
Rwanda (2005–2015)	0.2156	0.0289	0.13	0.2161	0.0286	0.13	0.1139	0.0188	0.17	0.0376	−0.022	−0.59
Senegal (2005–2016)	0.2231	0.1388	0.62	0.2841	0.1925	0.68	0.1624	0.1611	0.99	0.2944	0.078	0.26
Togo (1998–2014)	0.0883	0.1533	1.74	0.0943	0.3759	3.99	0.0477	0.1345	2.82	0.0567	0.0815	1.44
Zambia (2002–2014)	0.0204	0.1218	5.97	0.0230	0.1462	6.36	0.0066	0.0363	5.50	0.0071	0.0462	6.51
Zimbabwe (1999–2015)	0.0566	0.0850	1.50	0.0621	0.0828	1.33	−0.0076	0.0311	−4.09	0.0569	−0.0097	− 0.17
West Africa	0.2807	0.1525	0.54	0.2996	0.2791	0.93	0.1320	0.2224	1.69	0.2000	0.1862	0.93
East and Central Africa	0.0585	0.1886	3.23	0.0668	0.1893	2.83	0.1435	0.1609	1.12	0.2789	0.1778	0.64
Southern Africa	0.1219	0.0673	0.55	0.1277	0.0771	0.60	0.0840	0.0662	0.79	0.1416	0.0388	0.27
SSA	0.1321	0.1076	0.82	0.1318	0.1629	1.24	0.0759	0.1548	2.04	0.1253	0.0573	0.46

Source: Author’s calculation based on DHS data

At the SSA level, inequality in the use of health facilities for deliveries and use of modern contraceptives reduced by about 18 and 44% respectively. On the contrary, inequality in skilled delivery assistance and 4+ antenatal visits increased by about 24 and 204% respectively. With the exception of 4+ antenatal visits which increased by 69% in West Africa, inequality in the use of the four reproductive health services reduced substantially in both West and Southern Africa. On the contrary, inequality in the use of the four reproductive health services increased substantially in East and Central Africa, except that for the use of modern contraceptives, inequality in utilisation reduced by 36%. The result at the individual country level is not different from the aggregates at the regional and sub-regional level. While in some countries, inequality either reduced or increased marginally, in others (Zambia, Togo, Niger, Guinea, Cote de’ Ivoire and Benin), inequality in the use of one or more of the four reproductive health services increased substantially. For example, in Zambia and Niger, inequality in the use of each of the four reproductive health services increased by over 500 and 275% respectively. In Guinea and Cote de’ Ivoire, inequality in the use of 4+ antenatal visits increased by over 700%, with inequality in the use of modern contraceptives increasing by over a 1000% in Guinea. It is also important to note that while inequality in the use of health facilities for delivery and skilled delivery assistance in Guinea and 4+ antenatal visits in Zimbabwe changed from being pro-poor to pro-rich, the use of modern contraceptives in Ghana, Malawi, Rwanda and Zimbabwe changed from being pro-rich to being pro-poor for the period under consideration.

Consistent with the earlier presentation, changes in inequality in 8+ antenatal visits and use of modern contraception by women in a union were also examined. The results (see Table 5) for both 8+ antenatal visits and use of modern contraceptives by women in union suggest a far lower magnitude of change between the two data point for each country compared to use 4+ antenatal visits and use of modern contraception by all women. For example, the use of the 8+ indicator reduced the change in inequality (number of times) between the two data points from 1.69 to 1.2 for West Africa, 1.12 to 0.2 for East and Central Africa, 0.79 to 0.1 for Southern Africa and 2.04 to 0.7 for SSA. The situation is not entirely different in the case of a switch from use of modern contraception by all women to only women in a union.

## Discussion

The paper set out to examine current levels and changes in utilisation and inequality in utilisation of reproductive health services in SSA. The results indicate that current levels of use of reproductive health services are high (above 60%) in some countries (18 out of 30 countries for health facility deliveries, 16 out of 30 for skilled delivery assistance and 10 out of 30 for 4 + anatenatal visits). In a few countries such as Chad, Ethiopia, Niger and Nigeria, utilisation levels were relatively low. In addition to current levels of utilisation, the results also suggest that (1) in majority of the countries, utilisation has improved over time, (2) utilisation of reproductive health services is concentrated among the rich in majority of the countries, (3) Inequality in the use of reproductive health services has increased over time in some countries and in some instances changed from marginally being pro- poor to being pro-rich. On the contrary, when antenatal visits is measured using 8+ visits as the benchmark, and use of modern contraception is restricted to women in union, the results changes as follows: (1) percentage utilisation of antenatal care reduces, inequality increases and changes in both utilisation and inequality in the use of antennal care reduces overtime (2) percentage utilisation of modern contraception by women in union increases compared to all women, with pro-rich inequality reducing and the magnitude of changes over time also reducing both for utilisation and inequality in utilisation.

The results presented exhibits specific patterns that may benefit from further discussion. First, countries that tend to have low levels of utilisation such as Chad, Ethiopia, Niger and Guinea are all relatively poor countries. This may be an indication of the influence of income in the utilisation of reproductive health services. There is a large SSA literature that has consistently suggested that income is a strong predictor of utilisation of reproductive health services [33, 34, 44, 45]. On the contrary, countries such as Benin, Comoros, Congo, Ghana, Lesotho, Malawi, Namibia, Rwanda and Zimbabwe have relatively high utilisation levels but are not necessarily better off in terms of income than those with relatively lower utilisation levels. This may reflect the fact that other factors (education, culture and availability and accessibility to health facilities etc) other than income significantly influence the utilisation of reproductive health services [34, 44].

Secondly, the relatively high utilisation rate in Southern Africa compared to East and Central Africa and West Africa may be due to differences in the structure of their respective economies. The economies of most Southern African countries are formal compared to their East and Central Africa and West Africa counterparts. For example, existing estimates suggest that South Africa, Botswana, Swaziland and Lesotho have informal sectors (untaxed economy) that ranges from 0 to 20%; Zimbabwe, Kenya, Uganda, Gabon and Cameroon with informal sectors ranging from 20 to 40% and mostly West African countries (Ghana, Mali, Nigeria, Senegal, Burkina Faso etc) have informal sectors above 40% [46]. Thus, we argue that a higher level of formality in the economy is likely to be positively correlated with access to income and opportunities that may enhance the ability of citizens to secure access to health facilities and therefore improve the utilisation of reproductive health services [34, 47]. With respect to change in utilisation over time, Rwanda seem to stand out. As earlier indicated, Rwanda is not considered as one of the countries in SSA that is better off using either the size of their GDP or GDP per capita. However, continuity in development programming and strong leadership over the last decade has resulted in strong and marked progress in several development outcomes including the use of reproductive health services.

The inequality results is consistent with earlier results from Ghana, Malawi and Mozambique, that suggest that the use of reproductive health services is pro- rich [19, 20, 40, 48]. In most of these papers, the authors argue that key contributors to inequality in the utilisation of reproductive health services are inequality in the distribution of income/household wealth, women’s education and access to and availability of health facilities within the population [17, 19, 49]. The positive aspect of the current results is that, there are several countries where consumption of one or more reproductive health services is increasing (Burkina Faso, Ethiopia, Ghana, Malawi, Rwanda, Kenya, Lesotho etc) with inequality also reducing. More importantly, in Rwanda, Ghana, Malawi and Zimbabwe, use of modern contraceptives have changed from being pro-rich to pro-poor. This contradict the income related inequality narrative where authors have suggested that in many regions or countries, growing incomes have not necessarily resulted in equitable distribution of incomes [3–7].

While pointing out that the inequality situation in health and access to health care in SSA is not as challenging as the evidence presented in the income inequality literature, it is equally important to emphasise that there are a couple of countries where inequality in the utilisation of reproductive health services is growing. In the specific case of Guinea, pro-poor inequality in health facility deliveries and skilled delivery attendance changed to pro-rich inequality between the two periods. This may be explained by the inverse equity hypothesis, which argues that at lower levels of coverage, inequality may continue to be high given that the wealthy may constitute those with better access to services, with the poor catching up only at higher levels of coverage [50]. There is therefore the need to pay greater attention to countries where coverage of reproductive health services is still low, since failure to address such challenges can equally undermine progress made over the last couple of years.

Even more important is the fact that a change from using 4+ to 8+ antenatal visits and restricting use of modern contraceptives to women in union, changes both utilisation and inequality dynamics of the two reproductive health services. Clearly, using the 8+ indicator shows much lower levels of utilisation and higher levels of inequality compared to using the 4+ indicator, which has been the benchmark for some time now and used in most official reports. The use of the 8+ indicator could have crucial ramifications for the ability of many SSA countries to achieve the health-related SDGs, given that adequate levels of appropriate antenatal care will influence the achievement of several of the health-related SDG targets. The key question is whether the 4+ antenatal visits benchmark will continue to be used or there will be a switch to the 8 +? A switch to the 8+ may mean the need for SSA countries to direct substantial investments to improve access to antenatal care given that at current levels of utilisation, the use of 8+ gives substantially lower rates of utilisation. On the contrary, increased utilisation and reduced inequality associated with the use of modern contraception restricted to women in union compared to all women suggest that women out of a union may have restricted access to modern contraception. Given however that adolescents constitute the bulk of women 15–49 who are not in a union, the results may be an indication that adolescents in SSA may have restricted access to modern contraceptives. This may mean the need for SSA countries to equally increase investments in securing access to modern contraception for adolescents which could also have significant implications for economic growth and development through the demographic dividends [28–30].

The results in this paper have some limitation that I will argue arises from the trade-off needed to keep the focus of the paper. For instance, the cross-country focus of the paper makes it difficult to look at a variable like use of modern contraception in detail and other family planning variables. Also, the use of geography as a basis for comparison may be problematic, given that some of the countries within the SSA region are far richer and in a different income bracket compared to the others. Also comparing countries with data collected at different point in time could be erroneous, especially if the time difference is large. Notwithstanding the limitations enumerated above, the findings of the study are valid and can be crucial in extending our understanding of the inequality literature in SSA.

## Conclusion

The chapter set out to examine current levels and changes in both utilisation and inequality in utilisation of reproductive health services in SSA. As already indicated the results are mixed. Although the levels of inequality in the utilisation of reproductive health services remain high and pro-rich in many SSA countries, the promising aspect of the current results is the fact that in several countries, inequality in utilisation of reproductive health services have declined at a time when utilisation is increasing. The improving levels of utilisation and inequality suggest that, unlike the income inequality narrative, something positive is happening with the use of health services, and that existing interventions may be working well in those countries where utilisation and inequality are both improving. It may therefore be important to examine most of these countries in detail to unearth lessons that can be used to improve the situation in other countries in the sub-region where progress has either been too slow or non-existent. In this regard, Rwanda could be a good example to study, given that it has a relatively higher utilisation level, with increasing utilisation and declining inequality, even though its per capita income is one of the lowest in SSA. Lessons from Rwanda in terms of its health policy and implementation strategy could be helpful in improving outcomes in many SSA countries.

Besides the positive results, it is also important to emphasise that declining utilisation and increasing inequality in some countries, especially among the poor must be a source of worry. This is because increasing inequality coupled with reduction in consumption of reproductive health services in some countries can work together to compromise and reverse the gains made in the last couple of years. It is therefore important that players in the global health arena pay special attention to these countries and evolve the right set of interventions and incentives to improve utilisation. This will be important in reducing the currently high levels of inequality especially in East and Central Africa and West Africa, where the levels of inequality in the use of reproductive health services are currently high. Notwithstanding the challenges, the results equally suggest that some interventions are working well in some countries. In the last 20 years, improvement in the use of reproductive health services in SSA has been substantial [51]. Best practices and policies in these countries could be adopted to possibly improve conditions in the worst performing countries. This could be essential given that analysis of DHS reports, suggest that changes in the use of reproductive health services in several SSA countries over the last 20 years have been substantial. This will be crucial in accelerating progress towards the SDGs, given that SSA still lags behind almost all the other developing regions (Middle East and North Africa - MENA, Latin America and the Caribbean – LAC, Europe and Central Asia, South Asia) both in utilisation and inequality in the use of the four reproductive health services. Even more important is the urgent need for increased investments to create better access to both antenatal care and modern contraception for all women, especially adolescent girls.

## Data Availability

The Data used for the study is publicly available and can be accessed upon request from Measure DHS website (www.dhsprogram.com)

## Footnotes

1 The countries and respective years for data collection are Angola- 2016, Benin- 2012, Burkina Faso- 2010, Burundi- 2010, Cameroon- 2011, Chad- 2015, Cote de’ Ivoire- 2012, Comoros- 2012, Congo- 2012, Democratic Republic of Congo- 2014, Ethiopia- 2016, Gabon- 2012, Gambia- 2013, Ghana- 2014, Guinea- 2012, Kenya- 2014, Lesotho- 2014, Liberia- 2013, Malawi- 2016, Mali- 2013, Namibia- 2013, Niger- 2012, Nigeria- 2013, Rwanda- 2015, Senegal- 2016, Sierra Leone- 2013, Tanzania- 2016, Togo- 2014, Zambia- 2014 and Zimbabwe- 2014.

2 The following are the 20 countries used for the change estimates Benin (2001–2012), Burkina-Faso (98–2010), Cameroon (1998–2011), Chad (2004–2015), CIV (1998–2012), Ethiopia (2005–2016), Gabon (2000–2012), Ghana (2003–2014), Guinea (1999–2012), Kenya (2003–2014), Lesotho (2004–2014), Malawi (2004–2016), Mali (2001–2013), Namibia (2000–2013), Niger (1998–2012), Nigeria (2003–2013), Rwanda (2005–2015), Senegal (2005–2016), Togo (1998–2014), Zambia (2002–2014), Zimbabwe (1999–2015)

3 What is considered as modern contraception as per this paper include: pill, UID, injections, diaphragm, jelly, foam, condoms, female sterilisation and Norplant.
